# Metal Halide Perovskite Heterostructures: Blocking Anion Diffusion with Single-Layer Graphene

**DOI:** 10.1021/jacs.2c12441

**Published:** 2023-01-17

**Authors:** Matthew P. Hautzinger, Emily K. Raulerson, Steven P. Harvey, Tuo Liu, Daniel Duke, Xixi Qin, Rebecca A. Scheidt, Brian M. Wieliczka, Alan J. Phillips, Kenneth R. Graham, Volker Blum, Joseph M. Luther, Matthew C. Beard, Jeffrey L. Blackburn

**Affiliations:** † National Renewable Energy Laboratory, Golden, Colorado80401, United States; ‡ Department of Chemistry, University of Kentucky, Lexington, Kentucky40506, United States; § Thomas Lord Department of Mechanical Engineering and Material Science, Duke University, Durham, North Carolina27708, United States

## Abstract

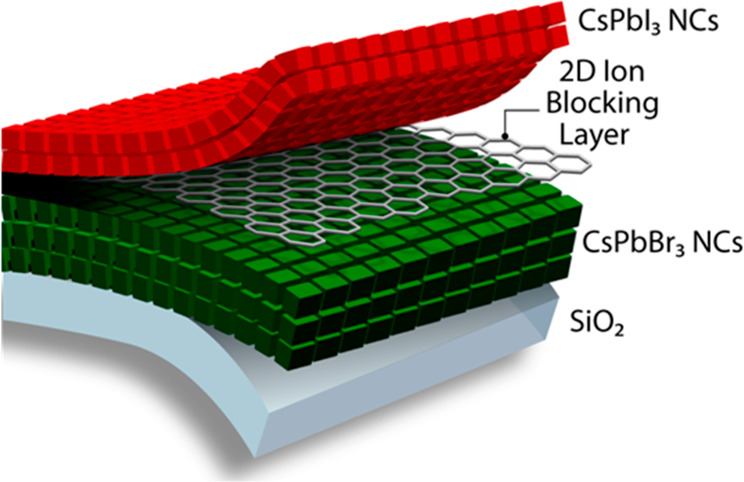

The development of metal halide perovskite/perovskite heterostructures is hindered by rapid interfacial halide diffusion leading to mixed alloys rather than sharp interfaces. To circumvent this outcome, we developed an ion-blocking layer consisting of single-layer graphene (SLG) deposited between the metal halide perovskite layers and demonstrated that it effectively blocks anion diffusion in a CsPbBr_3_/SLG/CsPbI_3_ heterostructure. Spatially resolved elemental analysis and spectroscopic measurements demonstrate the halides do not diffuse across the interface, whereas control samples without the SLG show rapid homogenization of the halides and loss of the sharp interface. Ultraviolet photoelectron spectroscopy, DFT calculations, and transient absorbance spectroscopy indicate the SLG has little electronic impact on the individual semiconductors. In the CsPbBr_3_/SLG/CsPbI_3_, we find a type I band alignment that supports transfer of photogenerated carriers across the heterointerface. Light-emitting diodes (LEDs) show electroluminescence from both the CsPbBr_3_ and CsPbI_3_ layers with no evidence of ion diffusion during operation. Our approach provides opportunities to design novel all-perovskite heterostructures to facilitate the control of charge and light in optoelectronic applications.

The ability to integrate semiconductors into heterostructure architectures is essential for enabling their use in a variety of optoelectronic applications. Advanced technologies typically integrate a variety of heterojunctions, homojunctions, etc., of conventional group IV and III–V semiconductors to enable a plethora of applications, such as photovoltaics, lasers, detectors, etc. In the case of metal halide perovskites (MHPs), heterostructures between various inorganic and organic semiconductors have enabled high-efficiency architectures, including photovoltaics,^1,2^ LEDs,^3^ and detectors/sensors.^4,5^ However, expanding upon these heterostructures and interfacial chemistries to include all perovskite/perovskite heterostructures, thereby mimicking conventional semiconductor heterostructures, has proved to be challenging.^6−14^

Specifically, facile anion diffusion between MHPs prevents APbX_3_/APbX′_3_ heterostructure growth, where X and X′ are different halides. When two perovskites of different halide compositions are deposited sequentially and in direct contact, the halide ions rapidly diffuse to form alloys instead of sharp interfaces.^15−17^ The resulting alloys lack the desirable properties of a sharp heterostructure interface, such as interfacial band alignment that facilitates control over carrier and energy transfer. This anion diffusion is so prominent that when a MAPbI_3_ (MA = methylammonium) film is simply mechanically stacked onto a MAPbBr_3_ film, the anions rapidly diffuse to form mixed compositions of MAPb(Br_3-*x*_I_*x*_).^18^ New approaches are needed to stabilize the composition of the heterostructures.

To solve the issue of halide mixing in MHPs, we looked to previous works on thin diffusion blocking materials. Previously, Bukola et al. showed single-layer graphene (SLG) acts as an ion diffusion blocking membrane in hydrogen pump cells, where protons pass through with high current densities; however, ions as small as potassium negligibly pass through the SLG.^19^ This demonstration suggested that SLG could also be an effective barrier for blocking the large halide anions found in MHPs. In a few recent MHP studies, various forms of graphene have been incorporated as an additive into the electrodes^20−23^ and transport layers^24−26^ of MHP photovoltaics to enhance the overall stability characteristics. However, there has been little success integrating SLG within an all-perovskite/perovskite heterostructure^27^ where the SLG serves as an ion diffusion blocking layer, and little is known regarding the degree to which such blocking layers impact optoelectronic properties and processes, such as charge/energy transfer. This is because most deposition conditions for graphene rely on polar solvents or require a high-temperature gas phase deposition, both of which degrade the MHP semiconductors.

To fabricate all-perovskite/perovskite heterostructures with SLG integrated as an ion diffusion blocking layer, we utilized a solvent-free transfer process of SLG to form stable CsPbI_3_/SLG/CsPbBr_3_ heterostructures.^19^ SLG grown on copper foil was pressed onto a heat release transfer tape followed by removal of the Cu foil by etching in ammonium persulfate, which left behind a single layer of graphene. The SLG/heat transfer tape was then hot pressed at 130 °C onto a CsPbBr_3_ nanocrystal (NC) film to cure the transfer tape and release the SLG from the tape and leave the SLG on top of the film (Figure 1a). Subsequently, a CsPbI_3_ NC film was deposited by spin coating on top of the SLG to complete the CsPbI_3_/SLG/CsPbBr_3_ heterostructure.

Time of flight–secondary ion mass spectrometry (ToF-SIMS) measurements verify the retention of the separate halide ions across the perovskite layers in the SLG-incorporated heterostructure.^28^ The CsPbI_3_ layer remains isolated at the top of the film while the CsPbBr_3_ layer remains at the bottom, with the SLG in between (compare red vs green shading in Figure 1b). In contrast, we find that the bromide and iodide anions rapidly mix throughout the film in the control sample of CsPbBr_3_/CsPbI_3_ deposited without the SLG (Figure 1c). Renderings of the individual ions and line profiles can be found in Figures S1 and S2. Cross-sectional SEM (Figure 1d) shows the heterostructure has a sharp interface between the perovskite films. Figure S3 shows the top view SEM images, Figure S4 shows AFM topography, and Figure S5 shows photographs of the heterostructure and control samples.

**Figure 1:**
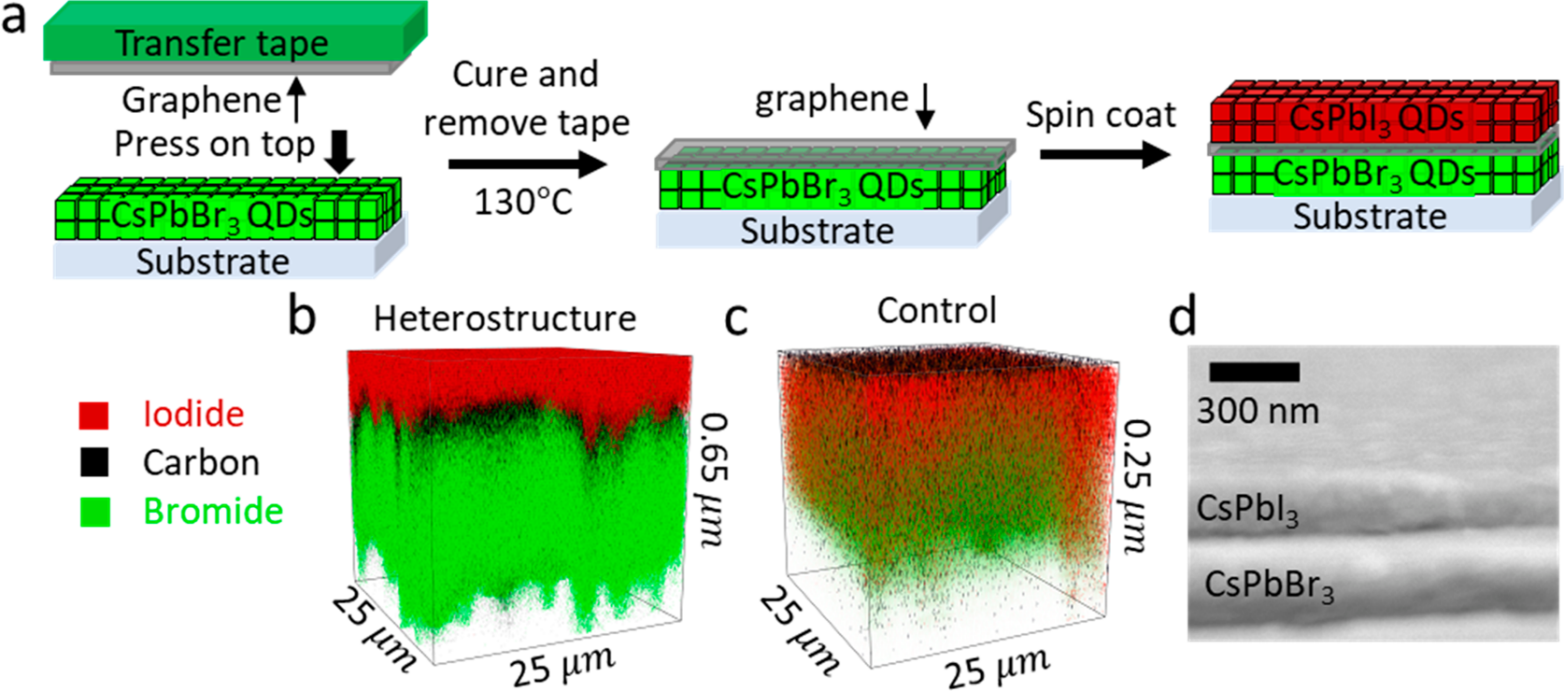
(a) Schematic of the single-layer graphene transfer process. TOF-SIMS 3D rendering (25 × 25 μm) of the (b) CsPbBr_3_/SLG/CsPbI_3_ heterostructure (depth, ∼650 nm) and (c) control without the SLG, i.e., direct contact of the perovskite layers (depth, ∼200 nm). (d) Cross section SEM image of the heterostructure.

To further confirm that the SLG sufficiently blocks halide diffusion, we used UV–vis absorbance and photoluminescence (PL) spectroscopies. A film of only CsPbBr_3_ NCs (Figure 2a, green traces) shows an absorption onset and PL emission at 520 nm, while a film of only CsPbI_3_ NCs (Figure 2a, red traces) shows an absorption onset and PL emission at 680 nm. When the NCs are deposited without SLG as a control sample, the absorbance onset and PL emission rapidly merge and exhibit an absorption onset and PL at 630 nm (Figure 2a, yellow trace), as expected when the halides mix and form CsPb(Br_3-*x*_I_*x*_).^17^ The halide mixing across the heterointerface is fast and is completed in the time scales required to transfer the samples for measurement (<30 min).

In contrast, for the CsPbBr_3_/SLG/CsPbI_3_ heterostructure, discrete absorbance and PL features persist (Figure 2a, black trace) and match that of isolated samples (Figure 2a, red and green traces). This is strong evidence that the heterostructure does not undergo halide diffusion across the SLG. Furthermore, we find that the PL peaks in a heterostructure sample stored at ambient conditions over 30 days show no clear evidence of shifting or change in the PL emission wavelengths (Figure 2b), thereby demonstrating that the SLG successfully blocks ion diffusion over long time scales.

**Figure 2:**
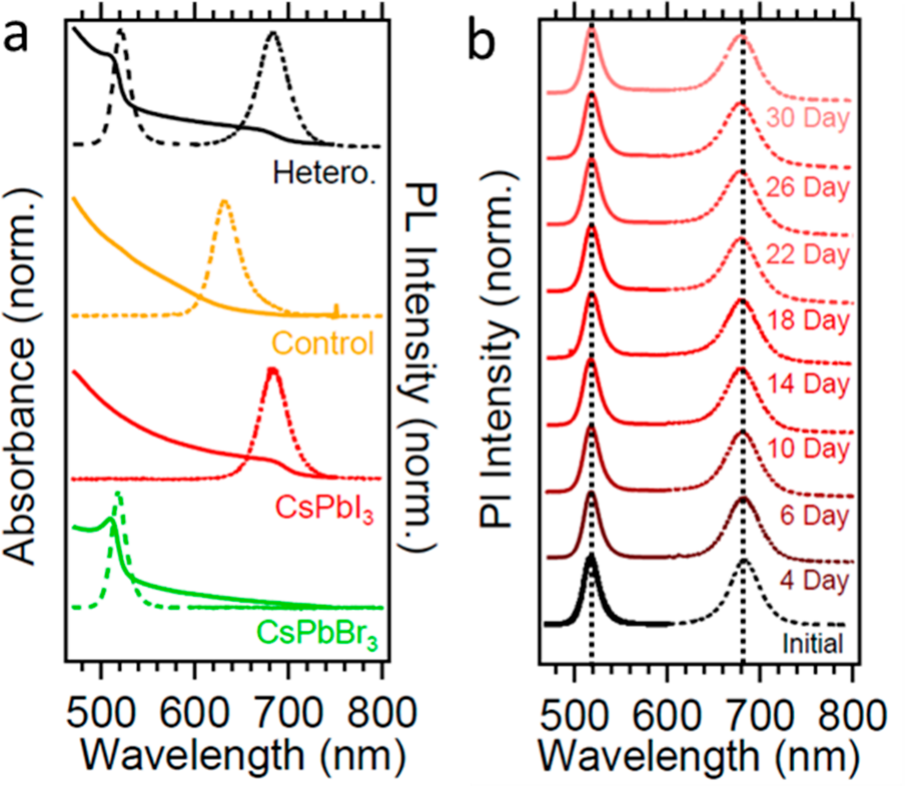
(a) Absorbance and PL spectra of control films of CsPbBr_3_, CsPbI_3_, and CsPbBr_3_/CsPbI_3_ deposited sequentially without the SLG (control), and of the heterostructure CsPbBr_3_/SLG/CsPbI_3_ (hetero.). (b) PL of the CsPbBr_3_/SLG/CsPbI_3_ heterostructure over the course of 30 days shows no evidence of halide intermixing, whereas the control sample is completely mixed almost instantaneously. Different dashed lines for spectra denote separate scans to probe the full wavelength range. Vertical dashed lines indicate initial positions of the two PL peaks.

The electronic properties of the CsPbBr_3_/SLG/CsPbI_3_ heterostructure were investigated with ultraviolet photoelectron spectroscopy (UPS). The alignment of the work functions of the MHP films with and without SLG (UPS spectra are shown in Figure S6) indicate that incorporating the SLG slightly raises the work functions of the NC films for both CsPbI_3_ and CsPbBr_3_ in a similar fashion, about 0.08–0.13 eV (compare right and left sides of Figure 3a). The measured work function of the SLG is slightly closer to vacuum than the VBs of both the CsPbI_3_ and CsPbBr_3_ (Figure 3a, left side).

From previous reports, the CsPbI_3_/CsPbBr_3_ interface is expected to exhibit a type I band alignment.^9^ Density functional theory (DFT) calculations corroborate this expectation. We used the FHI-aims code^29^ to relax the (010)-oriented slab supercells of CsPbI_3_ and CsPbBr_3_ interfaced with SLG at the CsX-terminated surface^30^ with the Perdew–Burke–Ernzerhof (PBE) semilocal density functional^31^ and a version of the Tkatchenko–Scheffler (TS) dispersion correction^32^ with a corrected van der Waals radius for Cs,^33^ a 3 × 1 × 3 *k*-point grid (SLG/CsPbI_3_ slab), and a 1 × 1 × 1 *k*-point grid (SLG/CsPbBr_3_ slab). Then, the band structures were calculated with the hybrid HSE06 functional^34,35^ with spin–orbit coupling^36^ and with 4 × 4 × 4 (CsPbBr_3_) *k*-point grids. The perovskite lattices were matched to the PBE+TS optimized lattice parameter of SLG (2.46 Å) by straining the CsPbI_3_ by 2.34% normal and 4.28% shear strain, and the CsPbBr_3_ was strained by 0.70% normal and 3.35% shear strain (strain reported relative to the respective PBE+TS-optimized relaxed perovskite lattices). Calculations of two other supercells with different strain indicate that the impact of this strain on the predicted band gap is negligible (Figure S10).

The band structure calculations suggest a type I band alignment (Figure 3b,c) for the heterostructre. Furthermore, the Dirac point of the SLG falls inside the respective band gaps of the perovskites and close to the VB. Figures S7 and S8 show the DFT-relaxed models of the slab CsPbX_3_/SLG structure, and Figure S9 shows the band structures of the bulk CsPbX_3_ without SLG.

**Figure 3:**
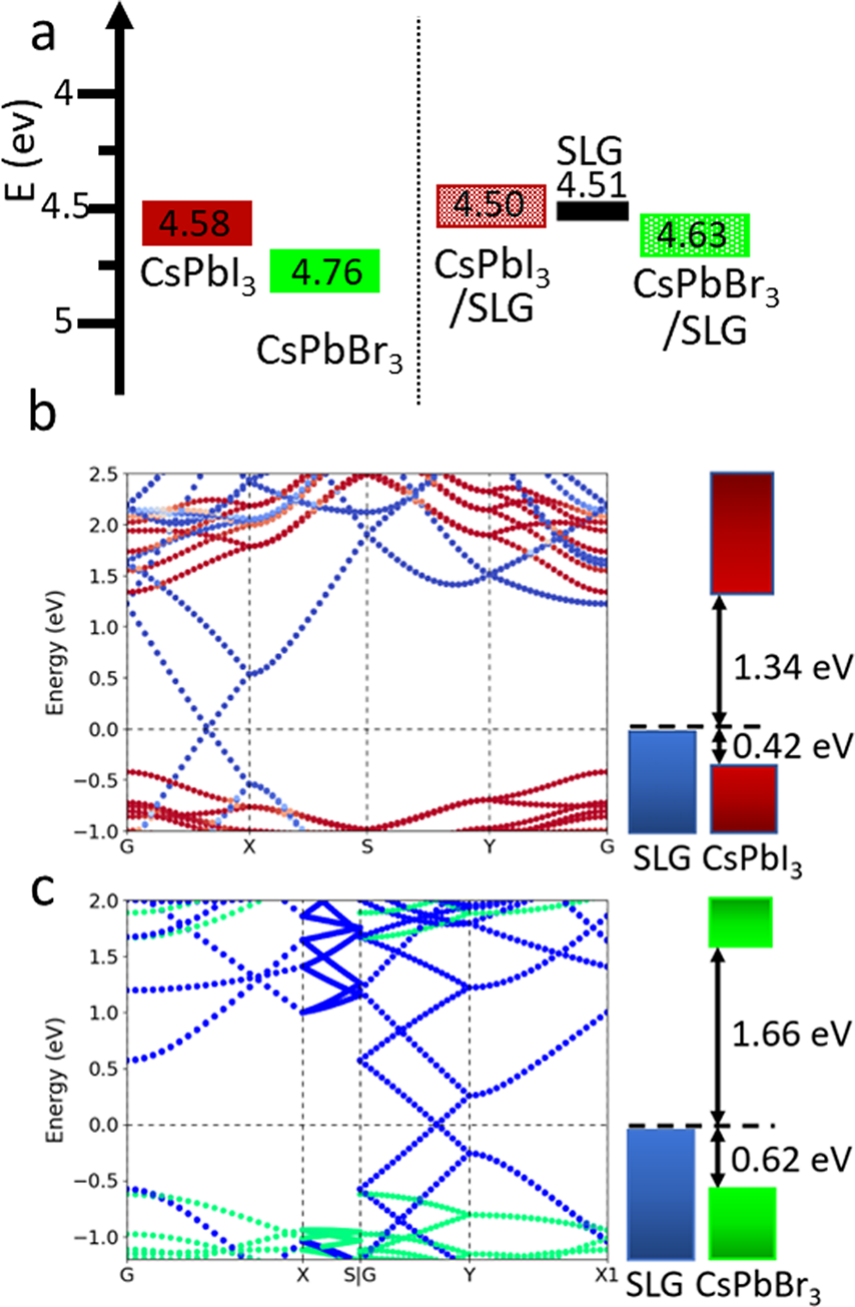
(a) Work functions versus vacuum (0 eV), as determined by UPS of the CsPbI_3_ and CsPbBr_3_ control samples and with the SLG showing the slight raising of the work function with SLG incorporation. The work function of the SLG is also shown. DFT band structure calculations of SLG incorporated with (b) CsPbI_3_ and (c) CsPbBr_3_. Schematics of the relaxed DFT structures are shown in Figure S6.

Transient absorbance spectroscopy (TAS) was used to probe the excited state dynamics of the heterostructure. Control experiments of individual CsPbI_3_ (Figure 4a red open circles) and CsPbBr_3_ (Figure 4b, green open circles) NC films integrated with SLG characterize the decay of the excited state when carriers reside in CsPbI_3_ or CsPbBr_3_. When integrated into the CsPbBr_3_/SLG/CsPbI_3_ heterostructure, carriers within CsPbBr_3_ decay faster relative to the control (compare green closed circles vs green open circles, Figure 4b). In contrast, carriers within CsPbI_3_, when incorporated into the heterostructure, decay slower relative to the control CsPbI_3_ (compare red closed circles to red open circles, Figure 3a).

The combination of the CsPbBr_3_ exhibiting a faster decay of the ground state bleach while the CsPbI_3_ decays slower suggests that photogenerated carriers can easily be transferred from the CsPbBr_3_ to the CsPbI_3_. We developed a simple transfer model to explain the data (black traces, Figure 3a,b). The decay rate within CsPbBr_3_ and CsPbI_3_ was characterized from the control experiments (solid black traces, Figure 4a,b). Then, the slower decay in the CsPbI_3_ and faster decay in the CsPbBr_3_ was simultaneously modeled by incorporating the transfer of carriers from the CsPbBr_3_ to the CsPbI_3_ (dashed black traces, Figure 4a,b). The transfer time was found via nonlinear least squares fitting to be ∼300 ps.

Our data suggest that the SLG does not act as a significant source of carrier recombination because in our model of the TA experiment we account for all photogenerated charge carriers either recombining or transferring from the CsPbBr_3_ to CsPbI_3_, i.e., no other decay channel was needed to model our data. In addition, we can conclude that both carriers are able to transfer (i.e., equivalent to energy transfer) in agreement with a type I band alignment because in the case of only charge transfer, we would expect the PL would quench in both films, which was not observed. Thus, the TAS results demonstrate the SLG does not hinder the electronic communication in between the perovskite layers.

To further probe the applicability of these heterostructures in a technologically relevant application, we fabricated LEDs with the CsPbBr_3_/SLG/CsPbI_3_ heterostructure. We observed stable electroluminescence from both CsPbBr_3_ and CsPbI_3_ (Figure S11). Because of processing conditions, the CsPbI_3_ NC layer was kept thin and, thus, exhibited a weaker electroluminescence intensity relative to the CsPbBr_3_. No mixing of the halides was observed, thereby confirming that the SLG enables optoelectronic-grade heterostructures.

**Figure 4:**
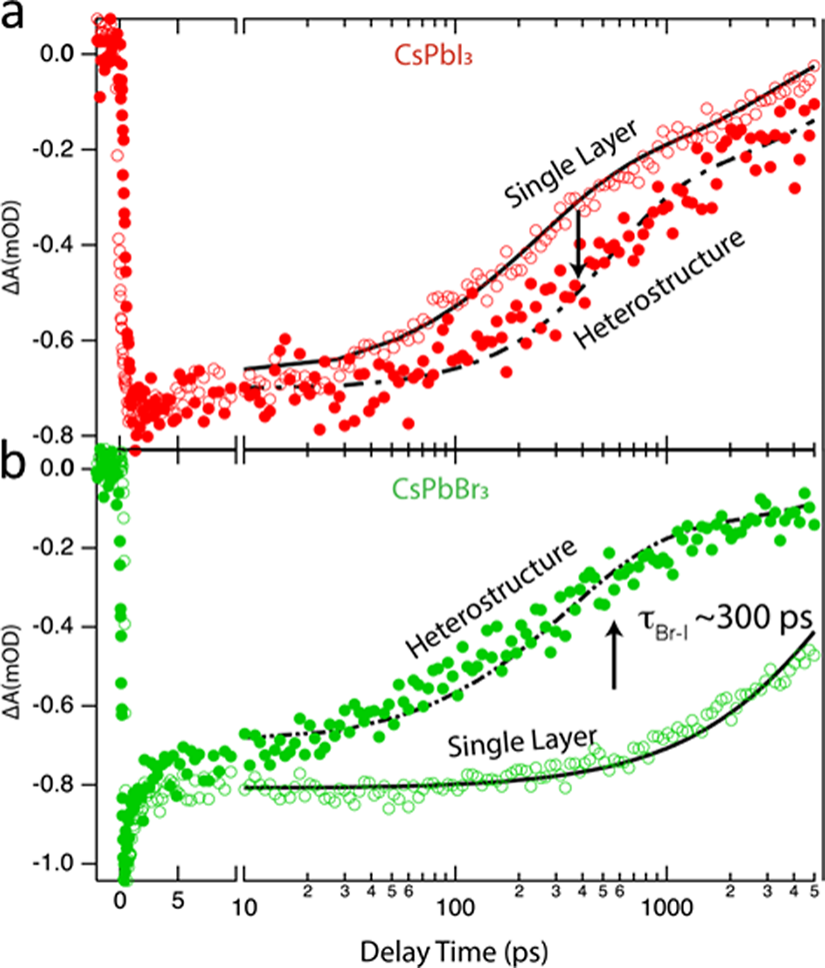
Transient absorbance spectroscopy of the CsPbI_3_ (*a*) and CsPbBr_3_ (b) control samples (with SLG integrated) in contrast to the CsPbBr_3_/SLG/CsPbI_3_ heterostructures. The black curve is the model for the single layer and heterostructure, where the CsPbBr_3_ has shorter lifetimes, while the CsPbI_3_ has longer lifetimes attributed to energy transfer.

We have developed a method to incorporate single-layer graphene at the interface between two MHP compositions and demonstrated that the SLG is an effective halide diffusion blocking layer; at the same time, the SLG does not degrade the optoelectronic properties of the perovskite semiconductors. The CsPbBr_3_/SLG/CsPbI_3_ heterostructures shown here show no signs of forming CsPb(Br_3-*x*_I_*x*_) alloys, which form almost immediately without the SLG blocking layer. The solvent-free and mild SLG deposition conditions developed here should be versatile for fabricating various perovskite/perovskite architectures that are similar to more advanced conventional semiconductor architectures. Furthermore, our initial results from UPS, DFT, and TAS indicate that the SLG has little effect on the heterostructure’s electronic behavior, which exhibits a type I band alignment that promotes energy transfer between the layers, thereby indicating electronic communication between the heterostructure components. This provides opportunities to integrate MHP semiconductors into targeted applications.

## Supporting Information Available

Experimental details, ToF-SIMS data, SEM images, photographs, UPS spectra, electroluminescence data, and DFT details (PDF)
