# Supplementary material for: Metal Halide Perovskite Heterostructures: Blocking Anion Diffusion with Single-Layer Graphene
Source: J Am Chem Soc. 2023 Jan 17;145(4):2052–7. doi: 10.1021/jacs.2c12441 (PMC9896553; doi:10.1021/jacs.2c12441)
Supplement: Supplementary file 1 — ja2c12441_si_001.pdf [file ja2c12441_si_001.pdf]

Supporting Information

Metal-Halide Perovskite Heterostructures: Blocking Anion Diffusion with Single Layer Graphene

Matthew P. Hautzinger[1], Emily K. Raulerson[1], Steven P. Harvey[1], Tuo Liu[2], Daniel Duke[3], Xixi Qin[3], Rebecca A. Scheidt[1], Brian M. Wieliczka[1], Alan J. Phillips[1], Kenneth R. Graham[2], Volker Blum[3], Joseph M. Luther[1], Matthew C. Beard[1], Jeffrey L. Blackburn[1]

[1]National Renewable Energy Laboratory, Golden, Colorado 80401, United States

[2]Department of Chemistry, University of Kentucky, Lexington, Kentucky 40506, United States

[3]Thomas Lord Department of Mechanical Engineering and Material Science, Duke University, Durham, North Carolina 27708, United States

1. Materials and Methods

Single layer CVD grown graphene on both sides of copper foil (35 μm thick) was purchased from ACS Materials, where the manufacture determined grain size was ~50 μm. Heat release tape was purchased from Semiconductor Equipment Corp. (21892-150MM, 9135 MS SS). Ammonium persulfate($(NH_4)_2(SO_4)_2$, 98%) cesium carbonate (Cs_2CO_3, 99%), lead iodide (PbI_2, 99.9985%), lead bromide ($PbBr_2$, 99.999%), hexane, octane, methylacetate, octadecene (90%), oleic Acid (90%), oleylamine (90%), Poly(3,4-ethylenedioxythiophene)-poly(styrenesulfonate), 3.0-4.0% in H_2O, high-conductivity grad (PEDOT:PSS); and PFN-DOF, Mw>10,000; were purchased from Sigma Aldrich.

Synthesis of $CsPbX_3$ nanocrystals

$CsPbX_3$ nanocrystals (NCs) were prepared following a reported method.[1] In a three-necked round-bottom flask, 2.50 mmol (0.814 g) of Cs_2CO_3, 2.5 mL oleic acid (OA), and 40 mL of octadecene (ODE) were degassed under a vacuum at 120°C for 1 hr followed by heating at 150°C until completely clear and gas was no longer evolved in the flask. In a 250 mL three necked flask, 3.25 mmol of PbX_2 (1.50 g PbI_2, 1.19 g $PbBr_2$) in 40 mL ODE was degassed at 120°C for 1 h under vacuum. Subsequently, 5 mL of oleic acid and 5 mL olelyamine were added to the reaction mixture. The temperature was then raised to 155°C ($CsPbI_3$) or 170°C ($CsPbBr_3$) followed by swift injection of 6.0 mL of the Cs-oleate precursor followed by immediately quenching the reaction with an ice bath. The NCs were then washed by adding 20 mL of dry methylacetate per 15 mL of reaction mixture followed by centrifuging at 8500 RPM for 5 min. The supernatant was discarded and the NCs were then redispersed in 15 mL of hexane per falcon tube (60 mL total) followed by centrifuging at 8000 RPM for 5 min. The supernatant was collected and stored overnight (~18 hr) in -18°C freezer to precipitate unreacted precursors. The precipitate was removed by centrifuging at 5000 RPM for 5 min and the supernatant was collected. The hexane was evaporated off under a stream of nitrogen and the NC solids were redispersed in 4 mL of octane total and diluted as needed.

$CsPbX_3$ NC films

All films were processed in a nitrogen glovebox. Glass or pre-patterned ITO substrates were sonicated in acetone then isopropanol for 10 min each, followed by a 15 min UV-ozone treatment. NC solutions were filtered through a 0.22 μm filter prior to use. The NC solution (30 μL) was deposited 3-5 s into 10 s spin cycle at 1000 rpm followed by 20s at 3000 rpm. The films were heated at 50°C to remove octane and used for heterostructure fabrication.

Graphene preparation

Single layer graphene on copper foil was cut into pieces slightly larger than the NC film substrate and pressed with 2000 kg of force onto the heat release tape. The tape/graphene compound was then soaked in 0.3 M aqueous $(NH_4)_2(SO_4)_2$ for roughly 18 h to completely dissolve the copper foil followed by thoroughly rinsing with DI water and drying with N_2 gas.

Heterostructure fabrication

$CsPbBr_3$ NC films were prepared as previously described. Graphene was then pressed onto the NC film with 600 kg of force for two minutes with both the top and bottom plate of the press pre-heated to 130°C. When removed, the tape's adhesive was entirely cured and could be removed from the films effortlessly. $CsPbI_3$ NCs were then spin coated as previously described on top of the graphene. For long term PL studies, a thin film of PMMA was spin coated on top of the heterostructure to protect the $CsPbI_3$ from moisture. For

ToF-SIMS measurements on the heterostructure, the $CsPbBr_3$ layer was deposited from a higher concentration to increase the thickness and improve the measurement quality.

LED Fabrication

ITO substrates were sonicated in acetone then isopropanol for 10 min each, followed by a 15 min UV-ozone treatment. In ambient atmosphere, PEDOT:PSS (3-4% in H_2O) was diluted 1:1 in DI H_2O then spin coated onto the substrate at 4000 RPM for 20 s, followed by annealing at 150°C for 20 minutes. Subsequently, the heterostructure was deposited as described in "Heterostructure Fabrication". Thereafter, PFN-DOF (5 mg/mL in chlorobenzene) was deposited by spin coating at 4000 RPM for 20 s. Au was evaporated (100 nm) as a top contact. A note: the spin coated deposition of PFN-DOF washed off a significant part of the $CsPbI_3$ film, leaving small amounts of material behind.

Absorbance and Photoluminescence (PL)

The absorbance spectra of the NC films and heterostructures were measured using ultraviolet–visible spectroscopy (Cary 6000i). PL spectra were acquired using a Horiba spectrophotometer equipped with a 405 nm laser with a collection time of 2 s, 600 line/mm grating, and a slit width of 50 μm. PL spectra were collected from 450-605 nm without a cutoff filter and 605-800 nm with a 550 nm cutoff filter to avoid the laser frequency doubling line.

Ultraviolet Photoluminescence Spectroscopy (UPS)

UPS was conducted in a PHI 5600 ultrahigh vacuum (UHV) system (~5×10^{-10} mbar) with a hemispherical electron energy analyzer. UPS spectra were obtained with an Excitech H Lyman-α lamp (E-LUX™121) with an excitation energy of 10.2 eV and a pass energy of 5.85 eV. A sample bias of -5V was applied to samples during UPS measurements.[2,3]

Transient Absorbance Spectroscopy

The transient absorption spectroscopy was performed with a home-built set up on a Ti:Sapphire amplifer (Coherent Astrella, 800 nm, ~60 fs pulse width, 1 kHz repetition rate). The output of the amplifier is split into two arms, one which pumps an optical parametric amplifier (Quantronix Palitra-Duo) and one which is used to generate white light continuum in a sapphire crystal. The probe is collected in an Ultrafast Systems Helios spectrometer. The pump wavelength used for all experiments was 450 nm with a pump energy of 16 nJ.

Scanning Electron Microscopy (SEM)

The morphologies of the perovskite films and cross-sectional structures of the heterostructure were investigated using a Hitachi S-4800 scanning electron microscope.

Time of flight secondary ion mass spectrometry (TOF-SIMS)

An ION-TOF TOF-SIMS V Time of Flight SIMS (TOF-SIMS) spectrometer was utilized for depth profiling and chemical imaging of the perovskite utilizing methods covered in detail in previous reports.[4] Analysis was completed utilizing a 3-lens 30kV BiMn primary ion gun. High mass resolution depth profiles were completed with a 30 KeV Bi_3^+ primary ion beam, (0.8pA pulsed beam current), a 50x50µm area was analyzed with a 128:128 primary beam raster. 3-D tomography and high-resolution imaging was completed with a 30KeV Bi_3^{++} primary ion beam, (0.1pA pulsed beam current), a 25x25µm area was analyzed with a 512:512 primary beam raster. Sputter depth profiling was accomplished with 1kV Cesium ion beam (6.5 nA sputter current) with a raster of 200×200 microns.

2. Figures S1-S11

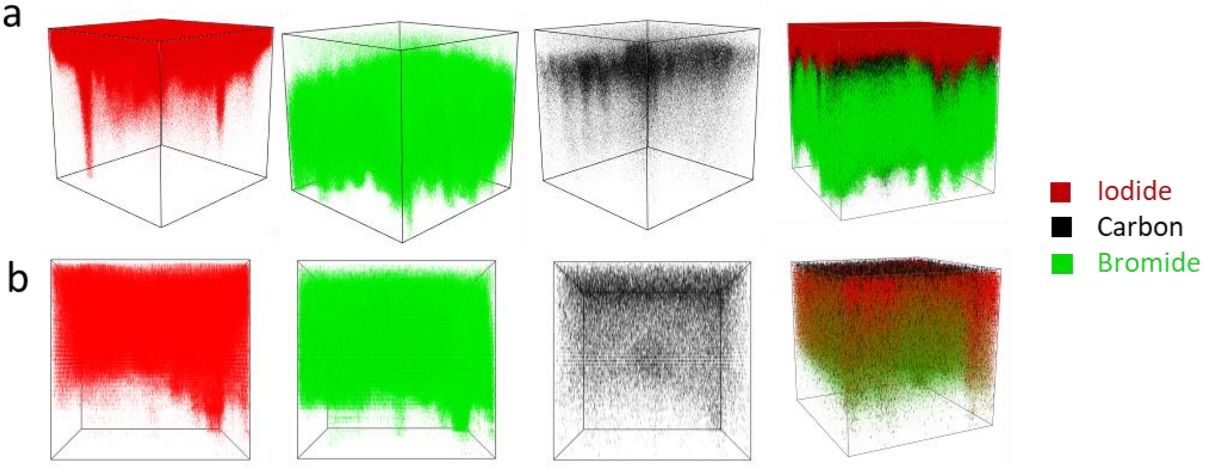

Figure S1. ToF-SIMS of individual ions and overlay of the (a) $CsPbBr_3$/SLG/$CsPbI_3$ heterostructure and (b) control $CsPbBr_3$/$CsPbI_3$ (i.e. no graphene/SLG). Each data set is 25x25 μm^2 in area and measurement depth of (a) ~650 nm and (b) ~ 200 nm. The graphene heterostructure in (a) was intentionally made thicker to enhance the resolution as described in the Materials and Methods section. Iodide rich particles on the surface or locally thicker parts of the film take longer to sputter through and thus are projected deeper into the film as it is profiled, causing the artificially deep "spikes' in the tomography data.

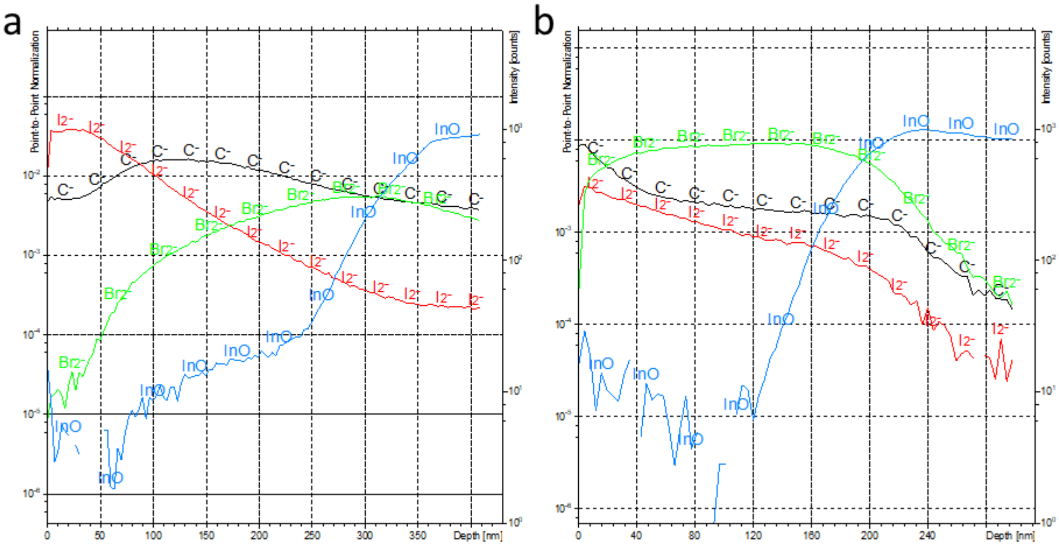

Figure S2. Line profiles of the heterostructure with (a) $CsPbBr_3$/SLG/$CsPbI_3$ heterostructure (b) control $CsPbBr_3$/$CsPbI_3$ (i.e. no graphene).

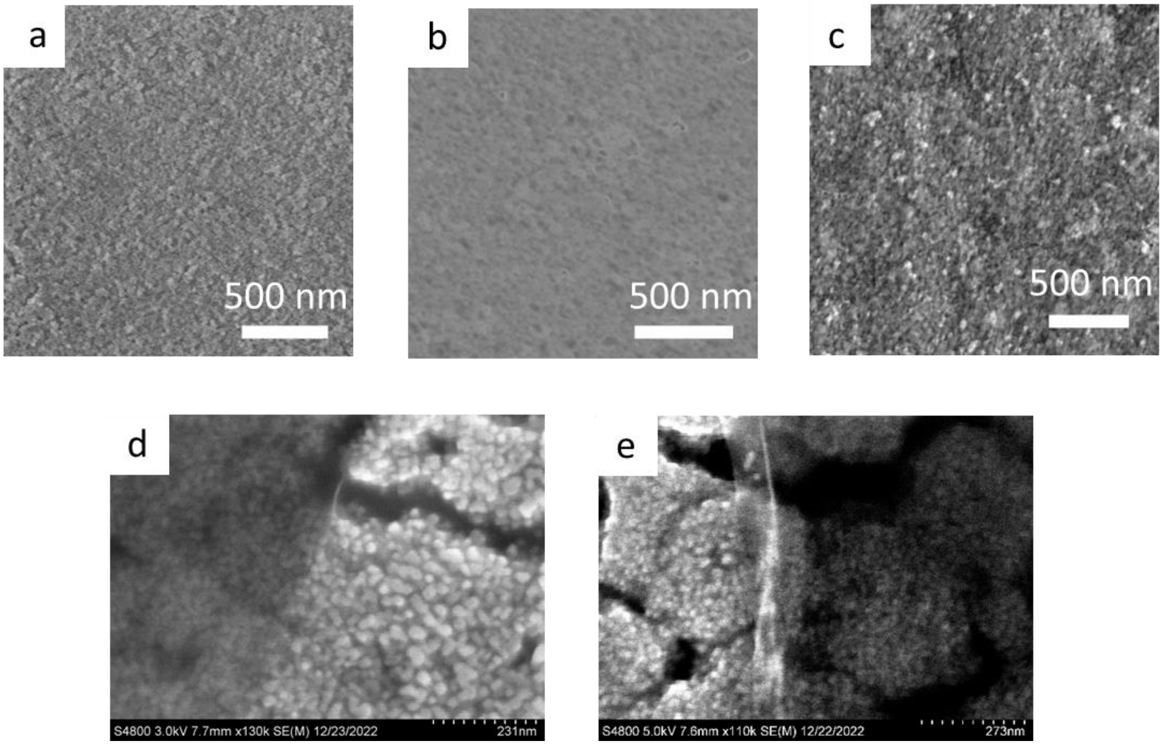

Figure S3. SEM micrograph of the (a) $CsPbBr_3$ NC film, (b) $CsPbBr_3$/SLG, and (c) $CsPbBr_3$/SLG/$CsPbI_3$ samples. Images (d) and (e) show $CsPbBr_3$/SLG samples specifically made for imaging, where only half the $CsPbBr_3$ was covered in SLG allowing imaging the SLG edges.

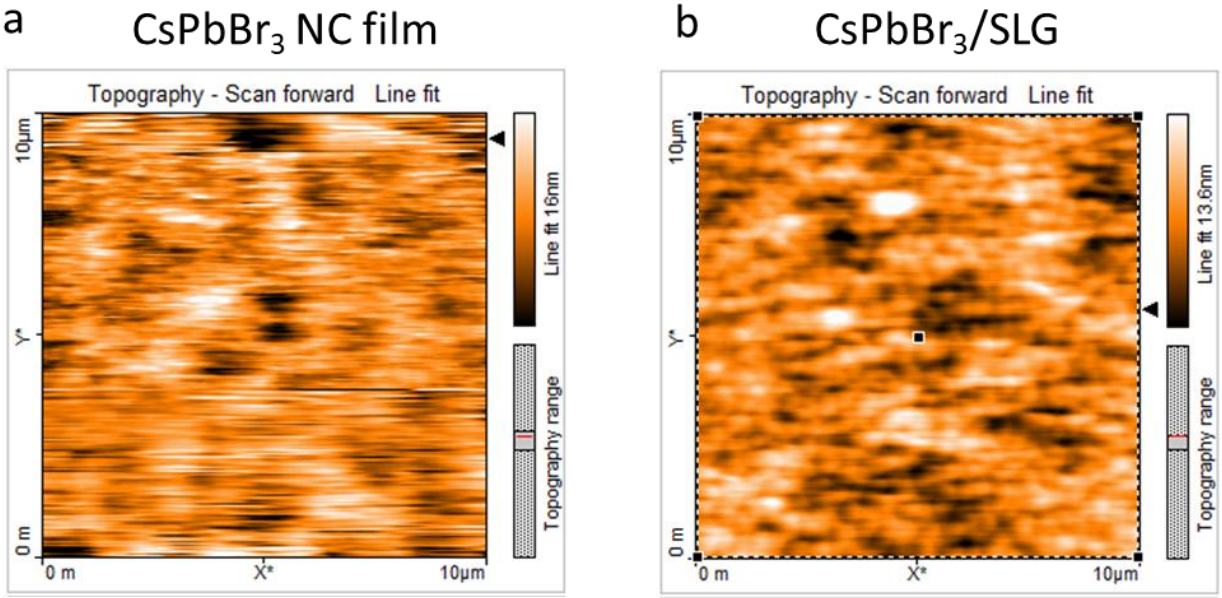

Figure S4. Images of AFM topography maps of the (a) $CsPbBr_3$ NC film (RMS = 3.0564 nm) and the (b) $CsPbBr_3$/SLG (RMS = 2.4345 nm). The topography maps show the film does not have significant changes before or after the graphene, with local deviations only up to 16 nm which is ~1-2 nanocrystals thick.

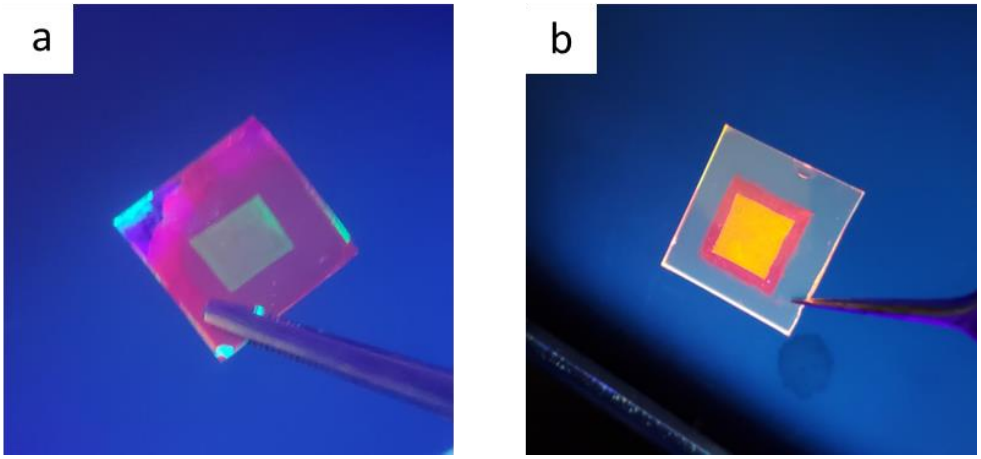

Figure S5. Photographs under UV illumination of the backside of the (a) $CsPbBr_3$/graphene/$CsPbI_3$ heterostructure and (b) sequentially deposited $CsPbBr_3$/$CsPbI_3$ film. In both films, the $CsPbBr_3$ was deposited onto the glass followed by scribing off material to provide a smaller region for the graphene transfer/consistency. Excess $CsPb(Br_{3-x}I_x)$ is present at the edges of the substrate from incomplete scribing.

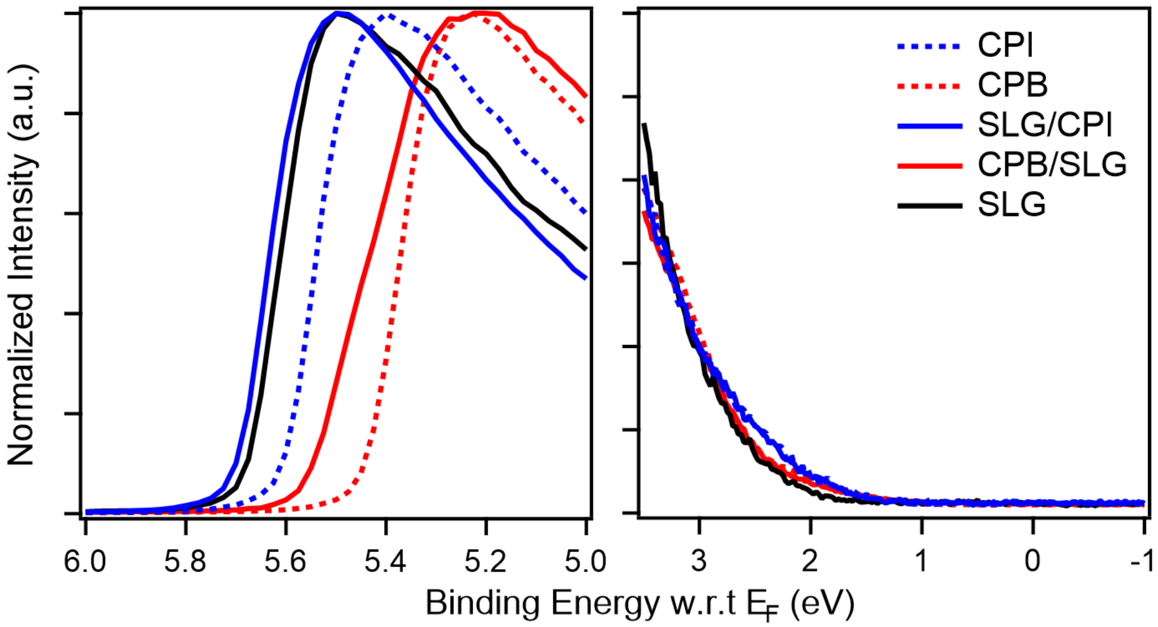

Figure S6. UPS spectra of the materials. CPI = $CsPbI_3$ NC film, CPB = $CsPbBr_3$ NC film, while CPX_SLG denotes samples with graphene present.

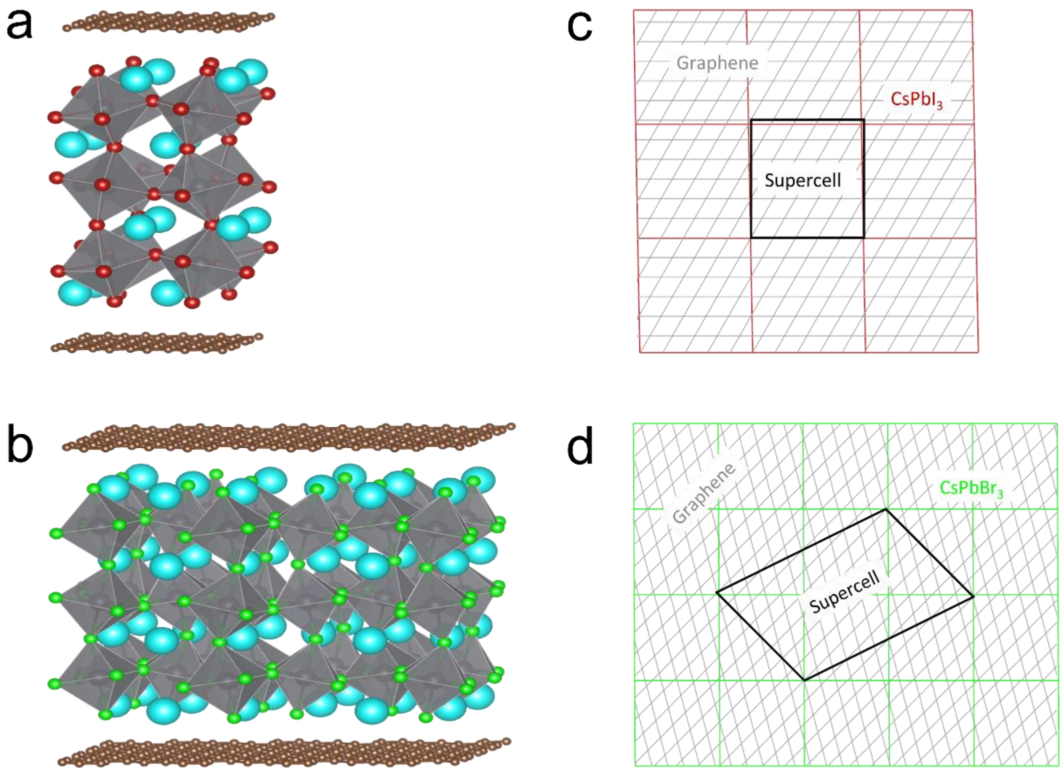

Figure S7. First slab structures relaxed with DFT-PBE+TS_alkaline with graphene and (a) $CsPbI_3$ and (b) $CsPbBr_3$ along with overhead views of the graphene/perovskite lattice combinations used to form the (c) $CsPbI_3$ and (d) $CsPbBr_3$ supercells. The $CsPbI_3$ supercell was strained by 5.49% normal and 8.15% shear strain compared to the experimental bulk structure[5], or 2.34% normal and 4.28% shear strain compared to the relaxed bulk structure. The $CsPbBr_3$ supercell was strained by 3.64% normal and 3.30% shear strain compared to the experimental bulk structure[6], or 0.70% normal and 3.35% shear strain compared to the relaxed bulk structure. In both supercells, the graphene experiences 0.36% normal and 0% shear strain compared to the experimental lattice, and no strain compared to the relaxed lattice. To retain the characteristics of the bulk material between the sheets of graphene, the positions of the atoms in the middle octahedral layer (consisting of one PbX_2 plane and two CsX planes) were fixed during the supercell relaxation. The perovskites interface with the graphene on the (010) plane, using the convention where the longest lattice vector is in the b-direction. The perovskites supercells are terminated by the CsX-plane because experimental work[7] suggests this termination is more likely. The PbX_2-terminated supercell was also simulated, and as expected, the band level alignment of the graphene within the perovskite bandgap did not match the UPS data as well as the CsX-terminated structure. All supercell and bulk relaxations were performed using PBE[8]+TS[9] with a corrected van der Waals radius for Cs[10,15], as implemented in FHI-aims all-electron code[11], with "intermediate" basis sets and numerical settings, and with 3×1×3 ($CsPbI_3$) and 1×1×1 ($CsPbBr_3$) k-point grids. To negate interaction between slabs, the graphene sheets were separated by 75Å of vacuum and a dipole correction was employed in the [010] direction. In the relaxed geometries, the moduli of residual forces on the atoms and, where applicable, on lattice parameters were below $5 \cdot 10^{-3}$ eV/Å. For both supercells, the band structures were calculated using the hybrid Heyd-Scuseria-Ernzerhof (HSE06) functional[12,13] with spin-orbit coupling[14] with 4×4×4 k-point grids with the hybrid exchange-correlation coefficient set to 0.25 and a screening parameter of 0.11 (Bohr radii)$^{-1}$.

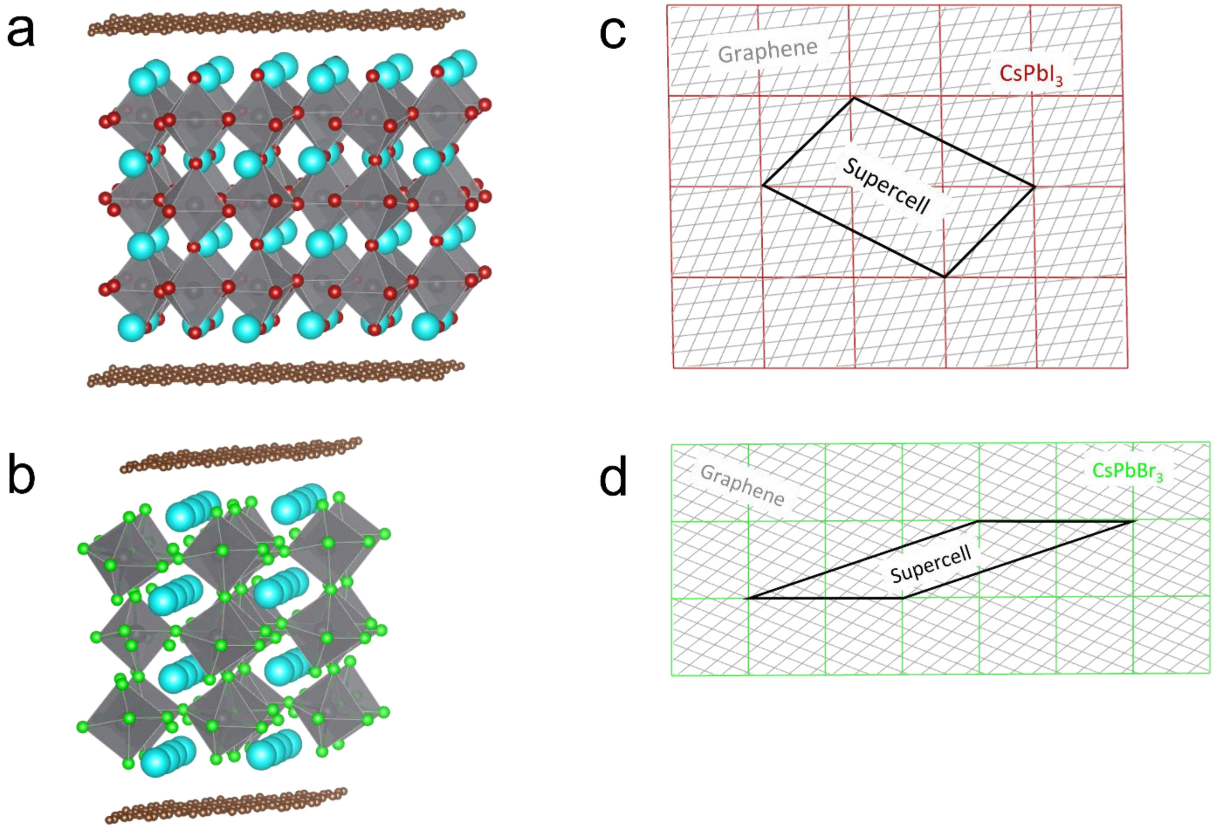

Figure S8. Second slab structures relaxed with DFT-PBE+TS_alkaline with graphene and (a) $CsPbI_3$ and (b) $CsPbBr_3$ along with overhead views of the graphene/perovskite lattice combinations used to form the (c) $CsPbI_3$ and (d) $CsPbBr_3$ supercells. All calculation parameters are the same as in Figure S7 except the k-point grids (1×1×1 for the SLG/$CsPbI_3$ slab and 2×1×2 for the SLG/$CsPbBr_3$ slab) and the strain on each supercell. The $CsPbI_3$ supercell was strained by 0.80% normal and 6.17% shear strain compared to the experimental bulk structure, or -2.22% normal and 2.06% shear strain compared to the relaxed bulk structure. The $CsPbBr_3$ supercell was strained by 2.99% normal and 7.17% shear strain compared to the experimental bulk structure, or 0.07% normal and 7.12% shear strain compared to the relaxed bulk structure.

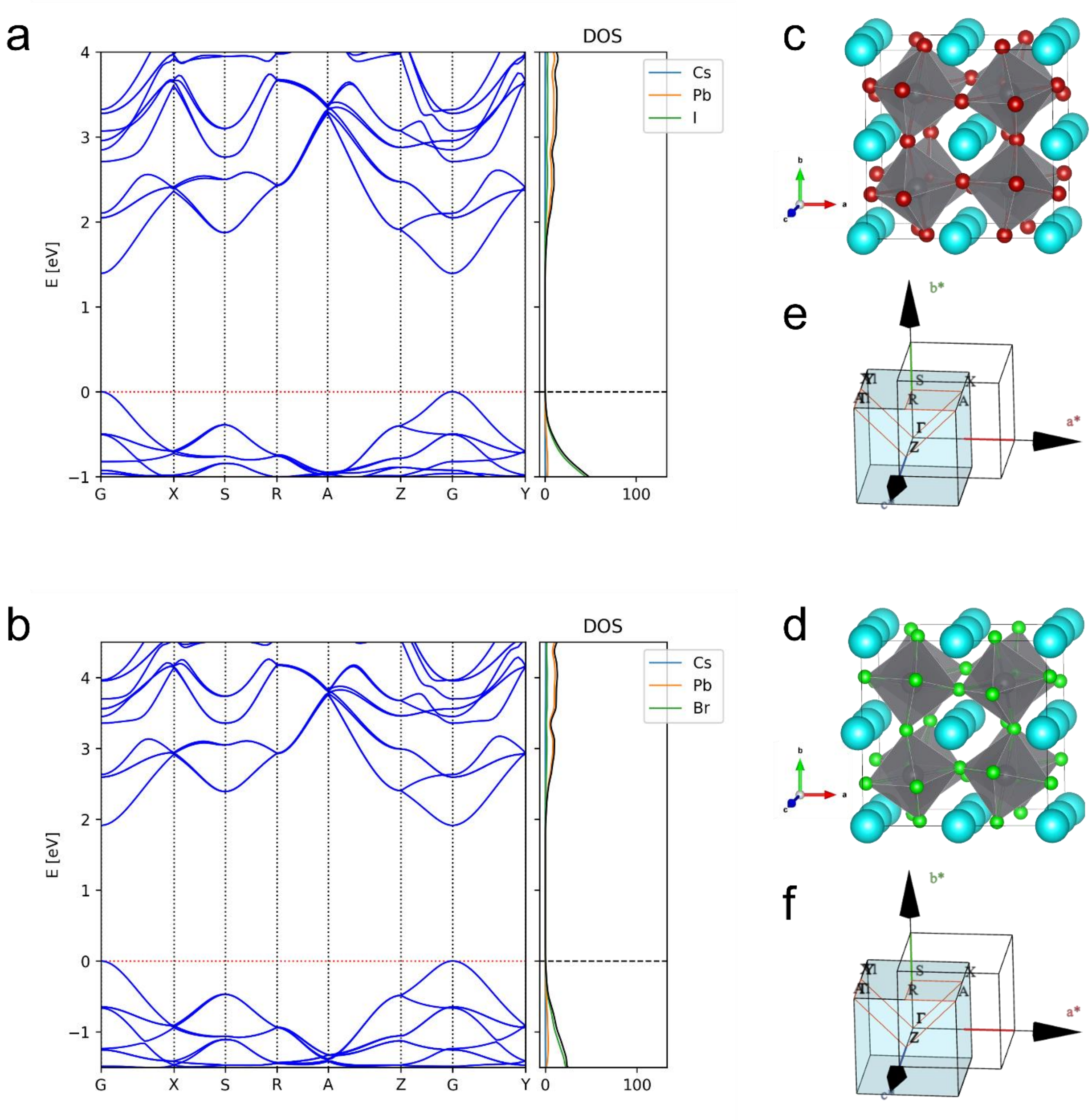

Figure S9. DFT band structure and density of states for bulk (a) $CsPbI_3$ and (b) $CsPbBr_3$ along with corresponding unit cell (c and e) and Brillouin zone showing the chosen k-path (d and f). Both structures were relaxed using the PBE+TS_alkali[15] functional, i.e., the original TS functional with a modified Cs van der Waals radius as described in Reference 15, and 4×4×4 k-point grids with "tight" basis sets as implemented in FHI-aims[11] and the convergence criterion forces and stresses set to 5E-3 eV/Å. The band structures were calculated with HSE06+SOC and 5×5×5 k-point grids with the hybrid exchange-correlation coefficient set to 0.25.

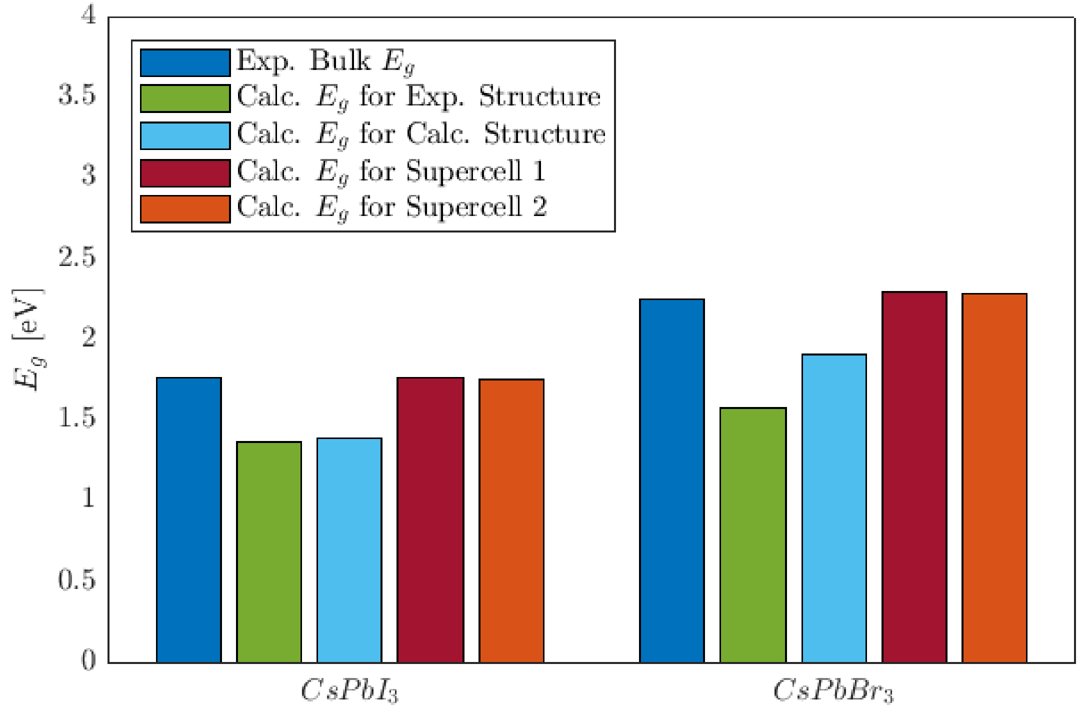

Figure S10. Comparison of experimental and calculated bandgaps for several structures, in particular the experimental bandgap determined from the absorbance spectra, the calculated bandgaps for the experimental bulk structures found in the literature[2,3], the calculated bandgaps for relaxed bulk structures, and the calculated bandgaps for the supercells described in Figures S6 and S7. The results show that although the DFT calculations underestimate the bandgap for the bulk structures, the calculated bandgaps match the experimental bandgaps for all supercells. Because both supercell pairs match each other, it is concluded that the strain applied to these supercells does not affect the bandgap in a way that is qualitatively significant for the purposes of this work.

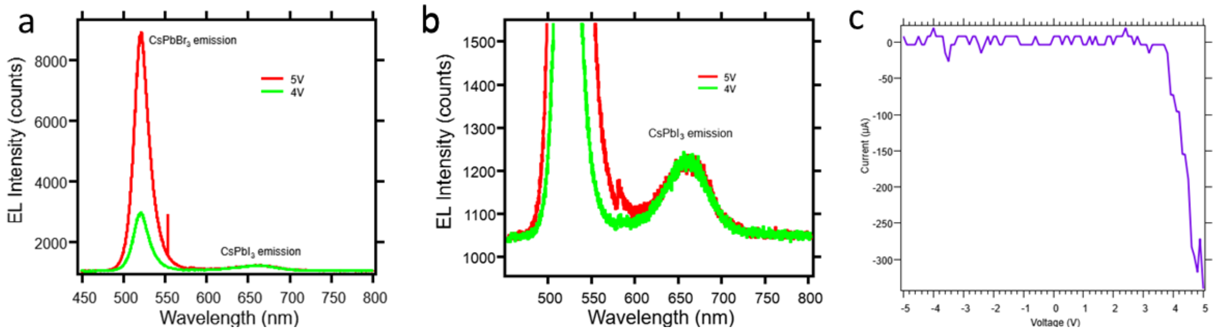

Figure S11. Electroluminescence of the ITO/PEDOT:PSS/$CsPbBr_3$/SLG/$CsPbI_3$/PFN-DOF/Au LED. The relatively low intensity of the $CsPbI_3$ emission is due to the processing conditions, as described in the methods section. The spin coated top layer of PFN-DOF washes away significant amounts of the $CsPbI_3$ film, leaving small amount of material behind, likely a reason for the weak $CsPbI_3$ emission. (c) shows the I-V characteristics of the device in the dark.

3. References:

(1) Protesescu, L.; Yakunin, S.; Bodnarchuk, M. I.; Krieg, F.; Caputo, R.; Hendon, C. H.; Yang, R. X.; Walsh, A.; Kovalenko, M. V. Nanocrystals of Cesium Lead Halide Perovskites (CsPbX3, X = Cl, Br, and I): Novel Optoelectronic Materials Showing Bright Emission with Wide Color Gamut. *Nano Lett.* **2015**, *15* (6), 3692–3696. https://doi.org/10.1021/nl5048779.

(2) Boehm, A. M.; Wieser, J.; Butrouna, K.; Graham, K. R. A New Photon Source for Ultraviolet Photoelectron Spectroscopy of Organic and Other Damage-Prone Materials. *Organic Electronics* **2017**, *41*, 9–16. https://doi.org/10.1016/j.orgel.2016.11.032.

(3) Boehm, A. M.; Liu, T.; Park, S. M.; Abtahi, A.; Graham, K. R. Influence of Surface Ligands on Energetics at FASnI3/C60 Interfaces and Their Impact on Photovoltaic Performance. *ACS Appl. Mater. Interfaces* **2020**, *12* (5), 5209–5218. https://doi.org/10.1021/acsami.9b17535.

(4) Harvey, S. P.; Messinger, J.; Zhu, K.; Luther, J. M.; Berry, J. J. Investigating the Effects of Chemical Gradients on Performance and Reliability within Perovskite Solar Cells with TOF-SIMS. *Advanced Energy Materials* **2020**, *10* (26), 1903674. https://doi.org/10.1002/aenm.201903674.

(5) Straus, D. B.; Guo, S.; Cava, R. J. Kinetically Stable Single Crystals of Perovskite-Phase CsPbI3. *J. Am. Chem. Soc.* **2019**, *141* (29), 11435–11439. https://doi.org/10.1021/jacs.9b06055.

(6) Stoumpos, C. C.; Malliakas, C. D.; Peters, J. A.; Liu, Z.; Sebastian, M.; Im, J.; Chasapis, T. C.; Wibowo, A. C.; Chung, D. Y.; Freeman, A. J.; Wessels, B. W.; Kanatzidis, M. G. Crystal Growth of the Perovskite Semiconductor CsPbBr3: A New Material for High-Energy Radiation Detection. *Crystal Growth & Design* **2013**, *13* (7), 2722–2727. https://doi.org/10.1021/cg400645t.

(7) Chen, Y.; Smock, S. R.; Flintgruber, A. H.; Perras, F. A.; Brutchey, R. L.; Rossini, A. J. Surface Termination of CsPbBr3 Perovskite Quantum Dots Determined by Solid-State NMR Spectroscopy. *J. Am. Chem. Soc.* **2020**, *142* (13), 6117–6127. https://doi.org/10.1021/jacs.9b13396.

(8) Perdew, J. P.; Burke, K.; Ernzerhof, M. Generalized Gradient Approximation Made Simple. *Phys. Rev. Lett.* **1996**, *77* (18), 3865–3868. https://doi.org/10.1103/PhysRevLett.77.3865.

(9) Tkatchenko, A.; Scheffler, M. Accurate Molecular Van Der Waals Interactions from Ground-State Electron Density and Free-Atom Reference Data. *Phys. Rev. Lett.* **2009**, *102* (7), 073005. https://doi.org/10.1103/PhysRevLett.102.073005.

(10) Fedorov, D. V.; Sadhukhan, M.; Stöhr, M.; Tkatchenko, A. Quantum-Mechanical Relation between Atomic Dipole Polarizability and the van Der Waals Radius. *Phys. Rev. Lett.* **2018**, *121* (18), 183401. https://doi.org/10.1103/PhysRevLett.121.183401.

(11) Blum, V.; Gehrke, R.; Hanke, F.; Havu, P.; Havu, V.; Ren, X.; Reuter, K.; Scheffler, M. Ab Initio Molecular Simulations with Numeric Atom-Centered Orbitals. *Computer Physics Communications* **2009**, *180* (11), 2175–2196. https://doi.org/10.1016/j.cpc.2009.06.022.

(12) Heyd, J.; Scuseria, G. E.; Ernzerhof, M. Hybrid Functionals Based on a Screened Coulomb Potential. *J. Chem. Phys.* **2003**, *118* (18), 8207–8215. https://doi.org/10.1063/1.1564060.

(13) Heyd, J.; Scuseria, G. E.; Ernzerhof, M. Erratum: Hybrid Functionals Based on a Screened Coulomb Potential [J. Chem. Phys. 118, 8207 (2003)]. *J. Chem. Phys.* **2006**, *124* (21), 219906. https://doi.org/10.1063/1.2204597.

(14) Huhn, W. P.; Blum, V. One-Hundred-Three Compound Band-Structure Benchmark of Post-Self-Consistent Spin-Orbit Coupling Treatments in Density Functional Theory. *Phys. Rev. Materials* **2017**, *1* (3), 033803. https://doi.org/10.1103/PhysRevMaterials.1.033803.

(15) Kim, Y.-H.; Song, R.; Hao, J.; Zhai, Y.; Yan, L.; Moot, T.; Palmstrom, A. F.; Brunecky, R.; You, W.; Berry, J. J.; Blackburn, J. L.; Beard, M. C.; Blum, V.; Luther, J. M. The Structural Origin of Chiroptical Properties in Perovskite Nanocrystals with Chiral Organic Ligands. *Advanced Functional Materials* **2022**, *32* (25), 2200454. https://doi.org/10.1002/adfm.202200454.
